# Inclusion of Quillaja Saponin Clarity Q Manages Growth Performance, Immune Response, and Nutrient Transport of Broilers during Subclinical Necrotic Enteritis

**DOI:** 10.3390/microorganisms11081894

**Published:** 2023-07-27

**Authors:** Candice E. C. Blue, Nima K. Emami, Mallory B. White, Staci Cantley, Rami A. Dalloul

**Affiliations:** 1 Department of Poultry Science, University of Georgia, Athens, GA 30602, USA; 2 School of STEM, Virginia Western Community College, Roanoke, VA 24015, USA; 3 Huvepharma, Inc., Peachtree City, GA 30269, USA

**Keywords:** necrotic enteritis, saponin, chicken, performance, tight junctions

## Abstract

Necrotic enteritis (NE) is an intestinal disease that results in poor performance, inefficient nutrient absorption, and has a devastating economic impact on poultry production. This study evaluated the effects of a saponin-based product (Clarity Q, CQ) during an NE challenge. A total of 1200 male chicks were randomly assigned to four dietary treatments (10 pens/treatment; 30 birds/pen): treatment 1 (NC), a non-medicated corn–soybean basal diet; treatment 2 (PC), NC + 50 g/metric ton (MT) of bacitracin methylene disalicylate (BMD); and treatments 3 (CQ15) and 4 (CQ30), NC + 15 and 30 g/MT, respectively. On the day (d) of placement, birds were challenged by a coccidia vaccine to induce NE. On d 8, 14, 28, and 42, performance parameters were measured. On d 8, three birds/pen were necropsied for NE lesions. On d 8 and d 14, jejunum samples from one bird/pen were collected for mRNA abundance of tight junction proteins and nutrient transporter genes. Data were analyzed in JMP (JMP Pro, 16), and significance (*p* ≤ 0.05) between treatments was identified by Fisher’s least significant difference (LSD) test. Compared to PC and NC, CQ15 had higher average daily gain (ADG), while CQ30 had lower average daily feed intake (ADFI) and feed conversion ratio (FCR). NE lesions in the duodenum were lower in CQ15 compared to all other treatments. On d 8, mRNA abundance of CLDN1, CLDN5, AMPK, PepT2, GLUT2, and EAAT3 were significantly greater in CQ30 (*p* < 0.05) compared to both PC and NC. On d 14, mRNA abundance of ZO2 and PepT2 was significantly lower in PC when compared to all treatments, while that of ANXA1, JAM3, and GLUT5 was comparable to CQ15. In summary, adding Clarity Q to broiler diets has the potential to alleviate adverse effects caused by this enteric disease by improving performance, reducing intestinal lesions, and positively modulating the mRNA abundance of various tight junction proteins and key nutrient transporters during peak NE infection.

## 1. Introduction

*Clostridium perfringens* alpha toxin (CPA) and the necrotic-enteritis-B-like pore-forming toxin (NetB) are produced by this anaerobic bacterium reported as the causative agent of necrotic enteritis (NE) in poultry [1,2,3]. NE is of economic importance to the industry as it has been estimated to cost approximately USD 6 billion in yearly losses [4,5]. To control pathogenic bacteria, sub-therapeutic doses of in-feed antibiotics have been used to improve broilers’ welfare and overall productivity [6,7]. However, with consumer demands for chickens raised without antibiotics and legislative restrictions, the poultry industry is looking to raise more flocks without antibiotic growth promoters (AGPs) [8,9]. However, with the removal of AGPs, incidences of NE have increased, leading the industry to find alternative strategies to improve bird health during this enteric challenge [10,11].

One strategy currently being studied to reduce enteric diseases is nutritional interventions like phytogenic-based feed additives [12,13,14,15]. Phytogenic feed additives (PFAs) are plant-derived bioactive compounds incorporated into animal feed or water [16,17,18]. They can improve productivity, available dietary energy, nutrient digestibility, innate immunity, and host disease resistance; alter the gut microbiota; decrease the prevalence of pathogens; and prevent bacterial colonization of the gastrointestinal tract [15,19]. *Quillaja saponaria* (QS) is classified as a PFA that is both fat- and water-soluble [20,21,22]. It is thought that the mechanism of action is their ability to penetrate the cell membrane affecting cellular permeability, which leads to leakage of critical molecules and ions from harmful bacteria [23,24,25]. However, there is still an ongoing need to alleviate the adverse effects caused by enteric diseases, as well as the need to better understand how these saponins improve performance and health. The central hypothesis was that Clarity Q has a positive effect on birds’ performance and response during this subclinical NE model. Therefore, this study was conducted to determine the effects of the saponin-based product Clarity Q on average daily gain (ADG), average daily feed intake (ADFI), feed conversion ratio (FCR), NE lesion scores, mRNA abundance of tight junction proteins, energy signaling pathways, and essential nutrient transport genes of broiler chickens during an NE challenge.

## 2. Materials and Methods

Day (d)-old Cobb male broiler chicks (n = 1200) were sourced from a local hatchery and randomly weighed and allocated to 4 treatment groups with each treatment consisting of 10 replicate pens and 30 birds per pen. Floor pens were ~1.22 m × 2.44 m and covered with fresh pine shavings as litter. The treatments included a negative control (NC) with birds fed a corn–soybean meal basal diet, a positive control (PC) fed the NC diet with bacitracin methylene disalicylate (BMD) at 50 g/metric ton (MT), and Clarity Q (CQ) added at 15 (CQ15) or 30 g/MT (CQ30). The diets were crumbled for the starter phase (d 0–14) and pelleted for the grower (d 15–28) and finisher (d 29–42) phases (Table 1). Chicks had access to feed and water ad libitum using a bucket-type feeder and a nipple drinker waterline. The light cycle was 24 h of light for the first three days, reduced to 23 h: 1 h for d 4–7, and then reduced to 18 h of light and 6 h of dark for the remaining duration of the trial. An automatic ventilation system was used to control the environment, and the temperature was maintained as follows: 32 °C for the first 3 days, then gradually reduced ~3 °C each week until it reached 23 °C at the start of week 4 where it remained constant. This study was conducted in accordance with the Institutional Animal Care and Use Committee guidelines.

### 2.1. Performance

Starting at placement, birds were monitored twice daily. Birds were weighed on d 8, 14, 28, and 42 on a per-pen basis. If dead birds were found, date, body weight, and cause of death were recorded. This procedure continued throughout the 42 d trial to record mortality/treatment for each phase, thus allowing for adjusting performance parameters such as body weight (BW), average daily gain (ADG), average daily feed intake (ADFI), feed conversion ratio (FCR), and adjustments for daily mortality.

### 2.2. Necrotic Enteritis Challenge and Lesion Scoring

As previously carried out in our lab, on the day of placement, feed and litter were sprayed with 10 × coccidiosis vaccine (Advent^®^; Huvepharma, Inc, Peachtree City, GA, USA) containing live oocysts of *Eimeria acervulina*, *E. maxima*, and *E. tenella* [26,27,28]. When coupled with the presence of *C*. *perfringens* spores in the barn environment, this leads to the development of an NE outbreak around one week after vaccine application [26,27,28]. On d 8, based on average body weight of each pen, 3 birds were randomly selected and euthanized via cervical dislocation, and the small intestine was removed to examine NE lesions. The duodenum and jejunum were scored separately by personnel blinded to the treatments based on a 0–4 scale system: 0 = no gross lesions, normal intestinal appearance; 1 = thin-walled or friable, gray appearance; 2 = thin-walled, focal necrosis, gray appearance, small amounts of gas production; 3 = thin-walled, sizable patches of necrosis, gas-filled intestine, small flecks of blood; 4 = severe extensive necrosis, marked hemorrhage, large amounts of gas in the intestine [29].

### 2.3. Total RNA Extraction and Reverse Transcription

On d 8 and d 14, one bird from each pen was humanely euthanized, and ~2 cm samples were immediately cut from the middle section of the jejunum, rinsed in cold PBS, snap-frozen in liquid nitrogen, and stored at −80 °C until analysis. The samples were homogenized by a bead mill homogenizer (TissueLyser II, Qiagen, Germantown, TN, USA), and total RNA was extracted with Trizol reagent following the manufacturer’s instructions (ZYMO Research, Direct-zol RNA Miniprep, Orange, FL, USA). Total RNA concentration was determined at optical density (OD) 260 (Nanodrop 1, Thermo Fisher, Waltham, MA, USA), and RNA purity was verified by evaluating the ratio of OD 260 to OD 280. After extraction, 2 μg of total RNA was reverse-transcribed into cDNA using the high-capacity cDNA Reverse Transcription kit (Applied Biosystems, Waltham, MA, USA) following the manufacturer’s protocol, and the cDNA was stored at −20 °C.

### 2.4. Quantitative Real-Time PCR

Quantitative real-time PCR (qRT-PCR) was performed using QuantStudio 3 (Applied Biosystems, Waltham, MA, USA) using PowerTrak Fast SYBR^TM^ Green Master Mix (Applied Biosystems, Waltham, MA, USA). The cDNA was diluted 1:20 in nuclease-free water, and 1.5 μL of the diluted cDNA was added to each well of a 96-well plate. Next, 8.5 μL of real-time PCR master mix containing 5 μL of Fast SYBR Green Master Mix (Applied Biosystems, Waltham, MA, USA), 0.5 μL each of forward and reverse primers (Table 2), and 2.5 μL of sterile nuclease-free water was added to each well for a final reaction volume of 10 μL. During the PCR reaction, samples were subjected to an initial denaturation phase at 95 °C for 120 s followed by 40 cycles of denaturation at 95 °C for 5 s and annealing and extension at 60 °C for 30 s. Each target reaction was performed in duplicate wells. Product specificity was confirmed by analysis of the melting curves produced by QuantStudio 3. mRNA abundances for tight junction proteins occludin (OCLN), claudin (CLDN) 1 and 5, zonula occludens (ZO) 1 and 2, and junctional adhesion molecules (JAMs) 2 and 3 as well as the nutrient transporter genes excitatory amino acid transporter 3 (EAAT3), glucose transporter (GLUT) 2 and 5, peptide transporter (PepT) 1 and 2, and sodium glucose transporter (SGLT) 1 were analyzed using glyceraldehyde-3-phosphate dehydrogenase (GAPDH) as an endogenous control. Cellular energy homeostasis pathways were also analyzed via AMP-activated protein kinase (AMPK), mammalian target of rapamycin (mTOR), and peroxisome proliferator-activated receptor gamma coactivator one alpha (PGC-1α), as well as mucin 2 (MUC2), pro-inflammatory gene tumor necrosis factor receptor-associated factor 3 (TRAF3), and anti-inflammatory gene Annexin (ANXA1). Average mRNA abundance relative to GAPDH for each sample was calculated using the 2^−ΔΔ^*^C^*^t^ method. The calibrator for each gene was the average ΔCt value from the negative control group of each corresponding day.

### 2.5. Statistical Analysis

Data were subjected to a one-way ANOVA using the JMP Pro 16 program, and a chi-squared test was used for lesion scores. Fisher’s least significant difference (LSD) test compared separated means when significant differences were noted. Statistical differences were considered significant at *p* ≤ 0.05.

## 3. Results

### 3.1. Performance Parameters

Performance data are presented in Table 3. Body weight displayed no significant difference between treatments throughout the 42 d trial. However, both CQ15 and CQ30 had numerically higher weights on d 28 (1381.27 and 1389.33 g/bird, respectively) and 42 (3165.79 and 3097.07 g/bird, respectively) compared to NC and PC. Average daily gain was similar for all treatments during the first two weeks. During d 0–28, the PC (55.59 g/bird) and CQ15 (55.81 g/bird) groups had significantly greater ADG compared to CQ30 (53.09 g/bird) but were comparable to NC. Average daily feed intakes in CQ30 and NC were lower compared to CQ15 but comparable to PC during d 9–14 and d 0–14. Overall (d 0–42), CQ30 had a numerically lower ADFI and FCR compared to all other treatments.

### 3.2. Mortality Rate

**Figure 1:**
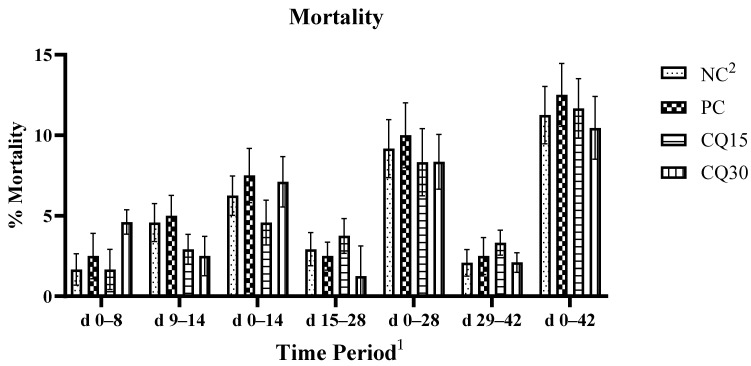
Effects of various inclusion rates of Clarity Q (CQ) on average mortality rate of broilers during a naturally occurring 42 d NE challenge. ^1^ Each bar represents the mean ± SE values of 10 replicate pens of 30 birds/pen. ^2^ Treatments included negative control (NC) as corn–soybean meal basal diet, positive control (PC) as NC + 50 g/MT of BMD, and NC + 15 or 30 g/MT of Clarity Q (CQ15 and CQ30, respectively).

The mortality rate is presented in Figure 1. There were no statistical differences amongst treatments; however, both CQ-supplemented treatments showed a reduction in percent mortality compared to NC and PC.

### 3.3. Necrotic Enteritis Lesion Scores

**Figure 2:**
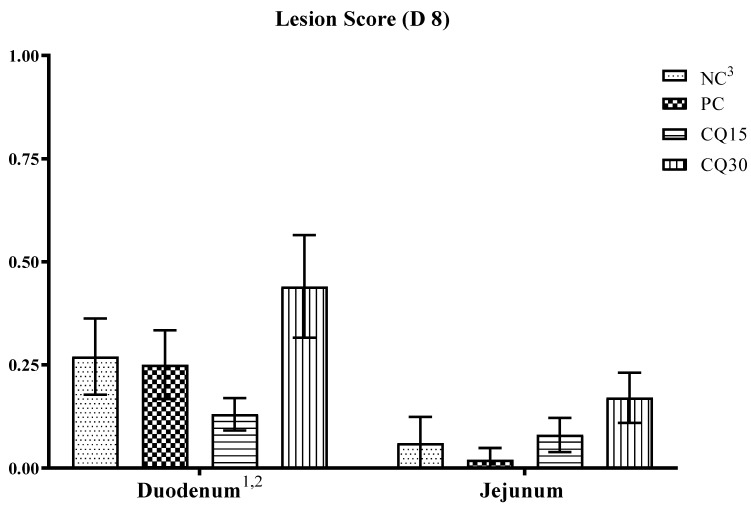
Effects of various inclusion rates of Clarity Q (CQ) on necrotic enteritis (NE) lesion scores on D 8 of broilers during a naturally occurring 42 D NE challenge. ^1^ Data represent the small intestinal sections (duodenum and jejunum) and mean value of 14 replicate pens of three birds/pen on day 8. ^2^ Each bar represents the mean ± SE values of 10 replicate pens of 30 birds/pen. ^3^ Treatments included negative control (NC): corn–soybean meal basal diet, positive control (PC) NC + 50 g/MT of BMD, and NC + 15 or 30 g/MT of Clarity Q (CQ15 and CQ30, respectively.

The effect of the dietary supplementation of the saponin-based product Clarity Q on necrotic lesions is presented in Figure 2. On d 8, although there were no statistical differences in lesion scores amongst treatments, CQ15 and PC supplements reduced NE lesions in the duodenum and jejunum, respectively, compared to all other treatments.

### 3.4. mRNA Abundance of Tight Junction Proteins, Cellular Energy Homeostasis Pathways, and Nutrient Transporters

**Figure 3:**
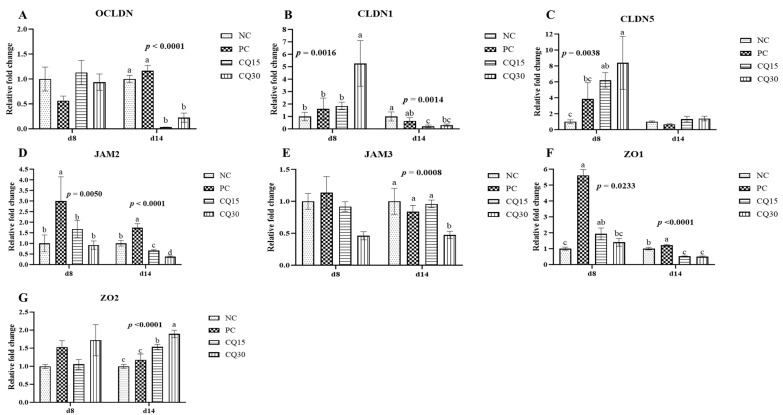
Relative mRNA abundance of tight junction proteins in the jejunum of broiler chickens on d 8 and d 14 during a 42 d NE challenge. Numbers with different letters (a–d) differ significantly (*p* < 0.05). Values are represented as *n*-fold difference relative to the calibrator (NC). (**A**–**G**) Each bar represents the mean ± SE values of 10 replicate pens of one bird/pen. (**A**–**G**) Occludin (OCLN), claudins (CLDN) 1 and 5, junctional adhesion molecules (JAMs) 2 and 3, and zonula occludens (ZO) 1 and 2. Treatments included negative control (NC) as corn–soybean meal basal diet, positive control (PC) as NC + 50 g/MT of BMD, and NC + 15 or 30 g/MT of Clarity Q (CQ15 and CQ30, respectively).

**Figure 4:**
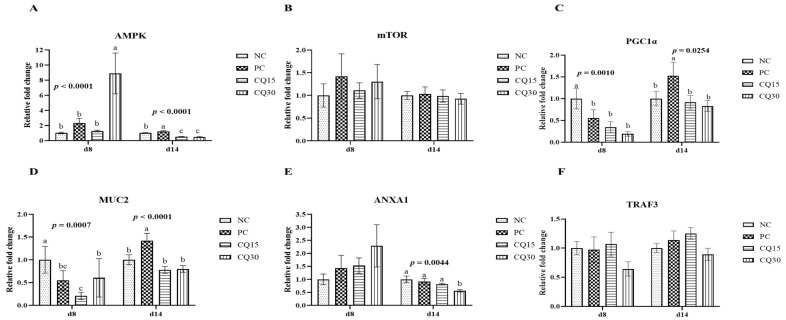
Relative mRNA abundance of signaling pathways and mucin in the jejunum of broiler chickens on d 8 and d 14 during a 42 d NE challenge. Numbers with different letters (a–c) differ significantly. (*p* < 0.05). Values are represented as *n*-fold difference relative to the calibrator (NC). (**A**–**F**) Each bar represents the mean ± SE values of 10 replicate pens of one bird/pen. (**A**–**F**) Adenosine monophosphate-activated protein kinase (AMPK), mammalian target of rapamycin (mTOR), peroxisome proliferator-activated receptor-gamma coactivator one alpha (PGC1α), mucin 2 (MUC2), annexin 1 (ANXA1), and TNF receptor 3 (TRAF3). Treatments included negative control (NC) as corn–soybean meal basal diet, positive control (PC) as NC + 50 g/MT of BMD, and NC + 15 or 30 g/MT of Clarity Q (CQ15 and CQ30, respectively).

**Figure 5:**
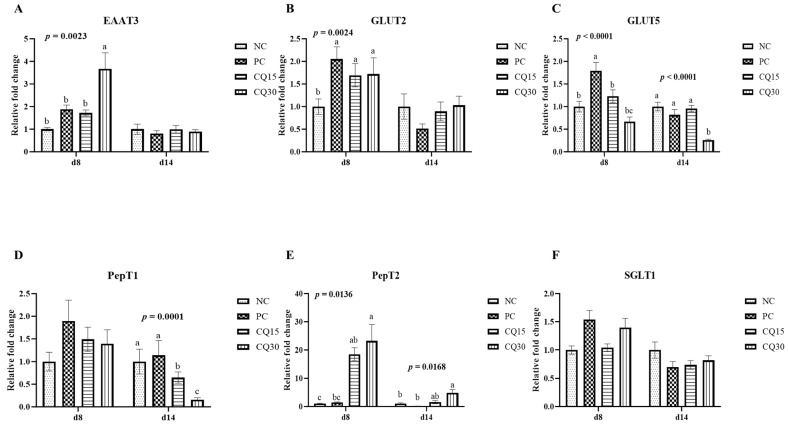
Relative mRNA abundance of nutrient transport genes of broiler chickens on d 8 and d 14 during a 42 d NE challenge. Numbers with different letters (a–c) differ significantly. (*p* < 0.05). Values are represented as *n*-fold difference relative to the calibrator (NC). (**A**–**F**) Each bar represents the mean ± SE values of 10 replicate pens of one bird/pen. (**A**–**F**) Excitatory amino acid transporter 3 (EAAT3), glucose transporter (GLUT) 2 and 5, peptide transporter (PepT) 1 and 2, sodium-dependent glucose co-transporter 1 (SGLT1). Treatments included negative control (NC) as corn–soybean meal basal diet, positive control (PC) as NC + 50 g/MT of BMD, and NC + 15 or 30 g/MT of Clarity Q (CQ15 and CQ30, respectively).

Figure 3, Figure 4 and Figure 5 show the effects of dietary supplementation of the saponin-based product Clarity Q on mRNA abundance of genes for tight junction proteins, markers of cellular energy homeostasis, and nutrient transporters. On d 8 (peak infection), CQ30 birds exhibited a significantly greater abundance of CLDN1 and CLDN5 (*p* = 0.0016 and *p* = 0.0038, respectively) compared to NC and PC, as well as AMPK compared to all other treatments. PC, CQ15, and CQ30 treatment groups exhibited greater abundance of EAAT3, GLUT2, and PepT2 compared to NC. GLUT5 mRNA abundance was greater in PC birds (*p* < 0.0001) compared to all treatments on d 8, while on d 14 NC, PC, and CQ15 had greater abundance compared to CQ30. Abundance of MUC2 and PGC1-α was significantly lower in CQ15 and CQ30 compared to NC on d 8 and PC on d 14. On d 14 (recovery phase), OCLDN, CLDN1, JAM2, and ZO1 showed lower abundance in CQ15 and CQ30 compared to NC and PC. However, mRNA abundance of ZO2 was significantly greater in CQ30 compared to all other groups. ANXA1 was significantly reduced in CQ15 and CQ30, while that of PepT1 was lower compared to NC and PC on d 14, and PepT2 was greater in CQ15 and CQ30 compared to NC and PC.

## 4. Discussion

Subclinical NE inflicts one of the greatest economic impacts on poultry production mostly due to reduction in feed efficiency [30]. In the current study, CQ30 showed a reduction in ADFI on d 9–14 and d 0–14 and caused a slight decrease in ADFI during the overall experimental period. This contributed to the overall better FCR in the CQ30-supplemented group, although these results were not statistically significant. Moreover, PC and CQ15 groups significantly increased ADG compared to CQ30 during d 0–28, showcasing that CQ supplementation could promote broiler performance similarly to an AGP. Similar studies have shown results supporting both positive and no effects of phytogenics on feed conversion under normal conditions or after a challenge with *C*. *perfringens* [12,31,32,33,34]. These varying effects on performance are not uncommon amongst studies and could be associated with the health of the flock, type of basal diet, and environmental conditions.

Damage to the intestinal mucosa is an important factor for *C*. *perfringens* colonization, and the presence of a coccidial infection is the most common causative factor facilitating *C*. *perfringens* pathogenesis [2]. Intestinal lesion scores are used to assess the severity of NE [26,27,35], and low scores are indicative of subclinical cases as presented herein. Although CQ15 displayed the lowest recorded lesions in the duodenum during peak infection (d 8), there was not a significant effect on lesions in either the duodenum or jejunum. A decrease in lesions could be an indication of a more intact, healthy, and functioning gut.

Tight junction proteins play an important role in gut integrity and homeostasis and are constantly remodeling in response to external stimuli in the gut lumen such as nutrients and commensal or pathogenic microbes [33,34,35]. The claudin family is a major component of tight junctions because it is an adhesion membrane protein, and several members are receptors for the bacterial toxins produced by *C*. *perfringens* [36]. Tight junctions are not the only first line of defense in maintaining the intestinal barrier; MUC2 is produced by goblet cells and is considered a biomarker of intestinal health because it reduces microbial adhesion to the mucosa [37,38]. Coccidia infection increases mucus production, which can contribute rapid proliferation of *C*. *perfringens* by providing protein-rich nutrients because of the damage caused to the host cell [39]. Our findings suggest that supplementing the diet with CQ increased the mRNA abundance of CLDN1 and CLDN5. Claudin 1 and occludin are markers for regulation of the tight junction paracellular permeability barrier, and they help seal the space between two enterocytes, avoiding the translocation of any harmful molecule from the intestinal lumen into the underlying tissue and bloodstream [36]. mRNA abundance of JAM3 and MUC2 was lower in the jejunum of supplemented groups opposite to that of ZO2 in the same tissues. This variation in abundance could be associated with better intestinal integrity and lower permeability in the CQ-supplemented groups which can also be demonstrated by the reduction in lesions.

Both AMPK and PGC1α are important in regulating energy metabolism within cells and play an essential role in intestinal health [35,40], while ANXA1 is important in the clearance of inflammation and restoration of mucosal homeostasis [41]. The current findings suggest that dietary supplementation of CQ can increase the abundance of AMPK during peak infection while slightly lowering the abundance of PGC1α during the recovery period. Since immune responses are energy demanding and tend to divert nutrients from growth resulting in reduced performance [27], a decrease in PGC1α could be the result of nutrients being directed towards growth rather than for mounting an immune response in the CQ-supplemented groups. The release of ANXA1 in the epithelial lining during peak infection could prevent the adverse effects caused by NE by inducing mucosal restoration and clearance of inflammation.

An intact epithelium prevents the entry of potential pathogens and results in optimal performance and increases the bird’s ability to better absorb and utilize nutrients [2]. Clarity Q at 30 g/MT expressed greater mRNA abundance of EAAT3, GLUT- 2, and PepT-2. Differences in the relative abundance of intestinal transporters such as PepT1, GLUT2, and EAAT3 are known to affect body weight, weight gains, and FCR in broilers [42]. Excitatory amino acid transporter 3 has also shown to be beneficial in initiating the amino-acid-dependent cell signaling in the mTOR pathway [39]. Nutrient transporters at the apical membrane of the small intestine are essential in moving nutrients into the enterocytes [43]. During dietary supplementation of saponins, SGLT1 and PepT1 have shown to increase cytoplasmic membrane recruitment, which has a positive effect on body weight and FCR in broilers [44]. In general, PepT1 is predominantly expressed in the small intestine, and its expression is upregulated in response to diet, malnourishment, and growth factors and could be a mechanism for using abundant resources or to compensate for the lack thereof [45,46]. This increased activity of disaccharides could also be attributed to an increased substrate presence at the apical membrane and can enhance the development of intestinal villi, which is important for efficient nutrient absorption and digestion [47].

In this subclinical NE challenge model, when broilers were supplemented with Clarity Q, the presented data showed a reduction in duodenal lesion scores on d 8 and a slightly improved FCR during the overall grow-out period. The results also showed a positive modulation in mRNA abundance of several tight junction proteins and nutrient transporter genes. As such, dietary supplementation of Clarity Q can potentially assist birds during an enteric disease challenge.

## Figures and Tables

**Table 1:** Composition of basal diets (as-fed basis, %) ^a^.

	Feeding Phase (Days)		
Ingredients (%)	Starter (1–14)	Grower (14–28)	Finisher (28–42)
Corn (7.81% CP)	59.53	64.12	65.70
Soybean meal (48% CP)	33.5	28.80	26.86
Soybean oil (9000 kcal/kg)	2.18	2.60	3.50
Dicalcium phosphate (18.5% P, 22% Ca)	2.05	1.92	1.70
Calcium carbonate (37% Calcium)	1.11	1.00	0.90
Sodium chloride	0.3	0.3	0.3
Sodium bicarbonate	0.07	0.07	0.05
DL-methionine (990 g/kg) ^b^	0.38	0.34	0.29
L-lysine hydrochloride (788 g L-Lysine/kg) ^c^	0.37	0.35	0.24
L-threonine (985 g/kg) ^d^	0.15	0.14	0.10
Vitamin/trace mineral premix ^e^	0.36	0.36	0.36
Calculated analysis (% unless specified)			
ME (kCal/kg)	3007	3087	3168
Crude protein	21.81	19.90	18.94
Total phosphorus	0.76	0.71	0.66
Available phosphorus	0.45	0.42	0.38
Calcium	0.90	0.84	0.76
Chlorine	0.33	0.33	0.29
Sodium	0.16	0.16	0.15
Potassium	0.85	0.77	0.73
Methionine	0.67	0.61	0.55
Methionine + cysteine	0.98	0.89	0.82
Lysine	1.32	1.19	1.05
Threonine	0.86	0.78	0.71
Linoleic acid	1.44	1.52	1.55
Dietary cation–anion balance (mEq)	194	174	170

^a^ The supplements were added to the basal mixes to provide the six experimental diets in every feeding period. ^b^ Rhodimet^®^ NP9, ADISSEO. ^c^ L-Lysine HCl, AJINOMOTO HEARTLAND. ^d^ FENCHEM Ingredient Technology. ^e^ Vitamins supplied per kg diet: retinol 3.33 mg, cholecalciferol 0.1 mg, α-tocopherol acetate 23.4 mg, vitamin K3 1.2 mg, vitamin B1 1.6 mg, vitamin B2 9.5 mg, niacin 40 mg, pantothenic acid 9.5 mg, vitamin B6 2 mg, folic acid 1 mg, vitamin B12 0.016 mg, biotin 0.05 mg, choline 556 mg. Minerals supplied per kg diet: Mn 144 mg, Fe 72 mg, Zn 144 mg, Cu 16.2 mg, I 2.1 mg, Se 0.22 mg.

**Table 2:** Sequences of primer pairs used for amplification of target and reference genes.

Target Gene	Primer Sequence	Accession No.
** Occludin (OCLN) **	F- CCGTAACCCCGAGTTGGAT R- ATTGAGGCGGTCGTTGATG	NM_205128.1
** Claudin 1 (CLDN1) **	F- GTGTTCAGAGGCATCAGGTATC R- TCAGGTCAAACAGAGGTACAA	NM_001013611.2
** Claudin 5 (CLDN5) **	F- AGGTGTCAGCCTTCATCGAC R- CCAGGATGGAATCGTACACC	NM_204201.1
** Junctional Adhesion Molecule 2 (JAM2) **	F- CTGCTCCTCGGGTACTTGG R- CCCTTTTGAAAATTTGTGCTTGC	XM_015299112.3
** Junctional Adhesion Molecule 3 (JAM3) **	F- CCAGAGTGTTGAGCTGTCCT R- AGAATTTCTGCCCGAGTTGC	XM_417876.6
** Zonula Occluden 1 (ZO1) **	F- GGAGTACGAGCAGTCAACATAC R- GAGGCGCACGATCTTCATAA	XM_413773
** Zonula Occluden (ZO2) **	F- GCGTCCCATCCTGAGAAATAC R- CTTGTTCACTCCCTTCCTCTTC	NM_204918.1
** AMP-activated protein kinase (AMPK) **	F- ATCTGTCTCGCCCTCATCCT R- CCACTTCGCTCTTCTTACACCTT	NM_001039603.1
** Mammalian target of rapamycin (mTOR) **	F- CATGTCAGGCACTGTGTCTATTCTC R- CTTTCGCCCTTGTTTCTTCACT	XM_417614.6
** Mucin 2 (MUC2) **	F- TTCATGATGCCTGCTCTTGTG R- CCTGAGCCTTGGTACATTCTTGT	XM_421035
** Annexin 1 (ANXA1) **	F- CTGCCTGACTGCCCTTGTGA R- GTTTGTGTCGTGTTCCACTCCC	NM_206906.1
** TNF Receptor-Associated Factor 3 (TRAF3) **	F- CTGAGAAAAGATTTGCCAGACCA R- CATGAAACCATGACACACGGG	XM_040672268.1
** Excitatory amino acid transporter 3 (EAAT3) **	F- GGTGAAGGCGGACAGGAA R- TGCTGAGCAGGAGCCAGTT	XM_424930.7
** Glucose transporter 2 (GLUT2) **	F- GAAGGTGGAGGAGGCCAAA R- TTTCATCGGGTCACAGTTTCC	NM_207178.1
** Glucose transporter (GLUT5) **	F- CCTCAGCATAGTGTGTGTCATCATT R- GGATCGGACTGGCTCCAA	XM_040689119.1
** Peptide transporter 1 (PepT1) **	F- GACAACTTTTCTACAGCCATCTACCA R- CCCAGGATGGGCGTCAA	NM_204365.1
** Peptide transporter 2 (PepT2) **	F- TGAAAAACCGCTCCCATCA R- TGTTCCGATGCCCAGTCAA	NM_001319028.1
** Sodium-dependent glucose cotransporter 1 (SGLT1) **	F- AGCATTTCAGCATGGTGTGTCT R- TGCTCCTATCTCAGGGCAGTTC	NM_001293240.1
** Glyceraldehyde 3-phosphate dehydrogenase (GAPDH) **	F- CCTAGGATACACAGAGGACCAGGTT R- GGTGGAGGAATGGCTGTCA	NM_204305.1

For each gene, the sequences for forward (F) and reverse (R) primers and the NCBI accession numbers used are listed.

**Table 3:** Effects of a phytogenic feed additive on average daily gain (g/bird), average daily feed intake (g/bird), and feed conversion ratio during a 42 d necrotic enteritis (NE) challenge.

Dietary Treatments ^1^					Statistics	
	NC	PC	CQ15	CQ30	SEM ^2^	*p*-Value
Days 0 to 8 *						
BW	175.22	176.23	180.16	176.02	2.09	0.3593
ADG	21.93	22.35	22.55	22.42	0.23	0.2752
ADFI	18.85	19.59	20.34	19.50	0.37	0.0625
FCR	0.86	0.88	0.90	0.87	0.02	0.3593
Days 9 to 14						
ADG	34.02	36.07	34.70	32.42	1.30	0.2724
ADFI	45.26 ^b^	46.11 ^ab^	48.51 ^a^	45.07 ^b^	0.88	0.0373
FCR	1.34	1.29	1.42	1.39	0.06	0.3687
Days 0 to 14						
BW	364.13	376.94	379.01	365.46	5.72	0.1698
ADG	31.03	31.93	31.56	31.55	0.56	0.7272
ADFI	34.16 ^b^	34.59 ^b^	36.60 ^a^	35.45 ^ab^	0.49	0.0081
FCR	1.10	1.09	1.16	1.12	0.02	0.1278
Days 15 to 28						
ADG	69.02	69.77	72.32	69.70	1.34	0.3370
ADFI	105.99	106.41	108.88	107.80	1.51	0.5229
FCR	1.54	1.53	1.51	1.55	0.01	0.1982
Days 0 to 28						
BW	1348.78	1370.61	1381.27	1389.33	22.61	0.1679
ADG	53.74 ^ab^	55.59 ^a^	55.81 ^a^	53.09 ^b^	0.78	0.0468
ADFI	75.14	76.90	78.00	75.06	0.90	0.0782
FCR	1.40	1.38	1.40	1.41	0.01	0.1705
Days 29 to 42						
ADG	111.73	112.65	114.45	111.66	3.02	0.9061
ADFI	177.38	182.62	182.93	175.35	2.25	0.0553
FCR	1.60	1.62	1.60	1.57	0.03	0.7096
Days 0 to 42						
BW	3062.56	3077.89	3165.79	3097.07	38.62	0.2662
ADG	54.86	55.13	56.13	55.06	1.37	0.9151
ADFI	83.35	83.86	86.01	83.26	2.09	0.7680
FCR	1.52	1.52	1.53	1.51	0.01	0.5241

* In each row, numbers with different letters (a–b) differ significantly. ^1^ Treatments included negative control (NC): corn–soybean meal basal diet, positive control (PC) NC + 50 g/MT of BMD and NC + 15, and 30 g/MT of Clarity Q (CQ15 and CQ30, respectively). ^2^ Standard error of the mean (SEM) represents values of 10 replicate pens of 30 birds/pen.

## Data Availability

Data are contained within the article.
